# Mechanistic Insights into Influenza A Virus-Induced Cell Death and Emerging Treatment Strategies

**DOI:** 10.3390/vetsci11110555

**Published:** 2024-11-10

**Authors:** Yuling Sun, Kaituo Liu

**Affiliations:** 1 Joint International Research Laboratory of Agriculture and Agri-Product Safety, The Ministry of Education of China, Yangzhou University, Yangzhou 225009, China; 2 College of Veterinary Medicine, Institute of Comparative Medicine, Yangzhou University, Yangzhou 225009, China

**Keywords:** influenza A virus, apoptosis, necroptosis, pyroptosis, PANoptosis, treatment

## Abstract

Influenza A virus (IAV) infection initiates a complex interplay of cell death modalities, including apoptosis, necroptosis, pyroptosis, and their integration, known as PANoptosis, which significantly impacts host immune responses and tissue integrity. These pathways are intricately regulated by viral proteins and host factors, contributing to both viral clearance and pathogenesis-related tissue damage. This review comprehensively explores the molecular mechanisms underlying these cell death processes in influenza infection. We highlight the roles of key regulatory proteins, such as ZBP1 (Z-DNA binding protein 1) and RIPK3 (receptor-interacting protein kinase 3), in orchestrating these responses, emphasizing the dual roles of cell death in both antiviral defense and tissue injury. Furthermore, we discuss emerging therapeutic strategies targeting these pathways, aiming to enhance antiviral efficacy while minimizing collateral tissue damage. Future research should focus on targeted approaches to modulate cell death mechanisms, aiming to reduce tissue damage and improve clinical outcomes for patients with severe influenza.

## Simple Summary

The influenza A virus (IAV) utilizes various mechanisms to manipulate host cell death pathways, impacting both the immune response and tissue damage during infection. This review discusses the intricate interplay between apoptosis, necroptosis, pyroptosis, and their integration, known as PANoptosis, highlighting how these pathways can either aid in viral clearance or exacerbate tissue injury. By understanding the dual nature of these cell death modalities, we aim to inform the development of targeted therapies that enhance antiviral responses while minimizing harm to the host.

## 1. Introduction

The influenza virus continues to pose a persistent public health threat, causing approximately 290,000 to 650,000 deaths annually during seasonal epidemics, with occasional pandemics, such as the H1N1 pandemic in 2009, highlighting its global impact [1]. The interplay between host immune responses and viral pathogenesis critically determines disease severity. Among the various cellular responses activated during influenza infection, programmed cell death mechanisms—including apoptosis, necroptosis, pyroptosis, and the emerging concept of PANoptosis—play critical roles in determining disease outcomes [2].

Apoptosis, often regarded as a regulated form of cell death, can either facilitate viral replication or promote viral clearance, depending on the specific context. Conversely, necroptosis and pyroptosis are characterized by inflammatory responses that can exacerbate tissue damage while attempting to contain viral spread. Recently, the concept of PANoptosis, which integrates features of apoptosis, necroptosis, and pyroptosis, has emerged as a crucial mechanism regulating host responses during viral infections, adding complexity to our understanding of the immune landscape.

Investigating the molecular pathways that govern these forms of cell death, particularly the roles of pivotal regulators, is essential for the development of targeted therapeutic strategies. This review aims to clarify the mechanisms of virus-induced cell death and discuss potential therapeutic strategies that could modulate these cell death pathways to mitigate tissue damage and improve clinical outcomes in patients with severe influenza.

## 2. Apoptosis Mechanisms and Regulation

Apoptosis, a highly regulated form of programmed cell death, is essential for maintaining tissue homeostasis and modulating immune responses. Apoptotic cells exhibit morphological characteristics, such as DNA fragmentation, chromatin condensation, pyknotic nuclei, and cell shrinkage (Table 1).

### 2.1. Mechanistic Insights into Apoptosis

**Figure 1:**
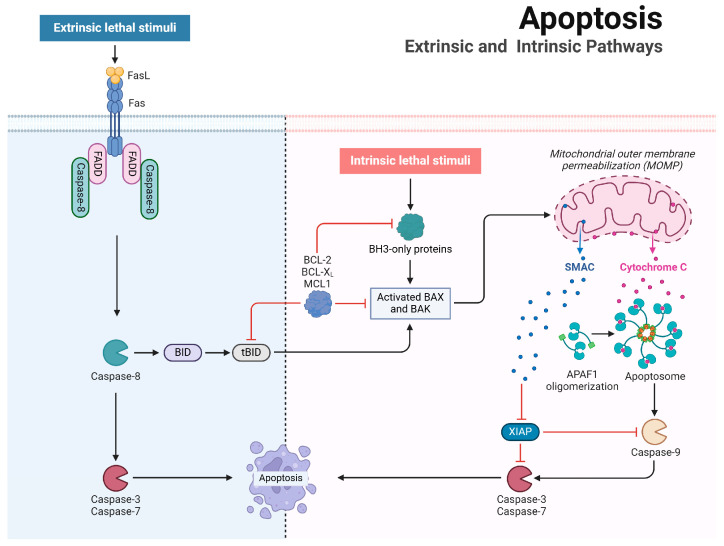
Mechanisms of intrinsic and extrinsic apoptotic pathways. The intrinsic pathway is initiated by various intracellular stress signals, leading to the oligomerization of BAX and BAK, which permeabilizes the mitochondrial outer membrane and facilitates the release of cytochrome c. This triggers the formation of the apoptosome and subsequent activation of caspase-9. In contrast, the extrinsic pathway is initiated by death receptors such as FAS, which, upon ligand binding, recruit adaptor proteins like FADD and activate caspase-8. Caspase-9 and caspase-8 executes apoptosis through the activation of caspase-3 and caspase-7. Caspase-8 can process BID, thereby linking the intrinsic and extrinsic pathways, while XIAP inhibits active caspases, thereby regulating the execution of apoptosis.

Apoptosis can be initiated via two primary pathways: the intrinsic (mitochondrial) pathway and the extrinsic (death receptor-mediated) pathway (Figure 1). The intrinsic pathway is triggered by various intracellular stress signals, such as DNA damage, accumulation of reactive oxygen species (ROS), and endoplasmic reticulum (ER) stress. These stressors activate pro-apoptotic BCL-2 family members like BAX and BAK, which oligomerize and permeabilize the mitochondrial outer membrane, facilitating the release of cytochrome c into the cytosol [3]. This process is tightly regulated by the balance between anti-apoptotic proteins (e.g., B-cell lymphoma 2 [BCL-2], BCL-XL, myeloid cell leukemia-1 [MCL-1]) and pro-apoptotic BH3-only proteins (e.g., BCL-2 antagonist of cell death [BAD], BH3-interacting domain death agonist [BID], p53 upregulated modulator of apoptosis [PUMA]) [4,5,6]. BH3-only proteins neutralize anti-apoptotic BCL-2 proteins, allowing BAX and BAK to activate. BAX translocates to the outer mitochondrial membrane, while BAK undergoes activation there. Together, they form oligomers that increase mitochondrial membrane permeability, releasing cytochrome c into the cytosol. Cytochrome c then binds to apoptotic protease-activating factor-1 (APAF-1), which oligomerizes to form the apoptosome [7,8,9,10]. The apoptosome, in the presence of deoxyadenosine triphosphate (dATP), recruits and activates initiator caspase-9, which subsequently cleaves and activates executioner caspases, such as caspase-3 and caspase-7, to carry out the dismantling of the cell [11]. Importantly, the balance between apoptosis and cell survival is further modulated by proteins such as X-linked inhibitor of apoptosis protein (XIAP), which binds to and inhibits active caspases, thus preventing the completion of apoptosis [12]. The pro-apoptotic protein SMAC, released from mitochondria, directly binds to inhibitor of apoptosis proteins (IAPs), thereby neutralizing their inhibition of caspases and promoting apoptosis.

In contrast, the extrinsic pathway is triggered by extracellular signals. Death receptors, such as Fas, TNF, and TRAIL receptors (DR4 and DR5), bind their respective ligands—Fas ligand, TNF-α, and TRAIL—resulting in the recruitment of adaptor proteins like FADD and TRADD, which initiate apoptotic signaling [13,14,15,16]. For example, Fas–FasL interactions cause Fas receptor clustering, facilitating the recruitment of the adaptor protein FADD. This leads to the activation of initiator caspase-8, forming the death-inducing signaling complex (DISC) [17]. Caspase-8 can then directly activate executioner caspases or cleave BID into its truncated form (tBID), thereby linking the extrinsic and intrinsic pathways through BAX/BAK-mediated mitochondrial outer membrane permeabilization [18].

Both the intrinsic and extrinsic apoptosis pathways converge on common substrates, notably caspase-3 and caspase-7, which are critical components of the cysteine protease cascade [11]. These caspases cleave various substrates, including poly(ADP-ribose) polymerase (PARP), Rho-associated protein kinase I (ROCK-I), ATPase 11A/C, and caspase-activated DNase (CAD). The cleavage of these proteins results in their functional inactivation, leading to the characteristic morphological changes of apoptosis [19].

### 2.2. Influenza A Virus Regulation of Apoptosis

Previous studies have shown that the influenza A virus (IAV) manipulates host apoptotic pathways through its viral proteins to optimize replication and survival (Table 2). A key player in this process is PB1-F2, which localizes to the mitochondria and induces mitochondrial membrane permeabilization, leading to cytochrome c release and activation of the intrinsic apoptotic pathway [20,21]. Another viral protein, M2, interacts with the ATG5/Beclin-1 complex to inhibit autophagosome fusion, indirectly promoting apoptosis [22]. The nucleoprotein (NP) of IAV also contributes to apoptotic regulation by stabilizing p53, a key pro-apoptotic factor, thereby amplifying apoptosis during infection [23]. In addition, NP can promote apoptosis by downregulating the expression of the host anti-apoptotic protein API5 [24], as well as reducing the interaction between CLU (Clusterin) and Bax, thus facilitating mitochondrial apoptosis [25].

**Figure 2:**
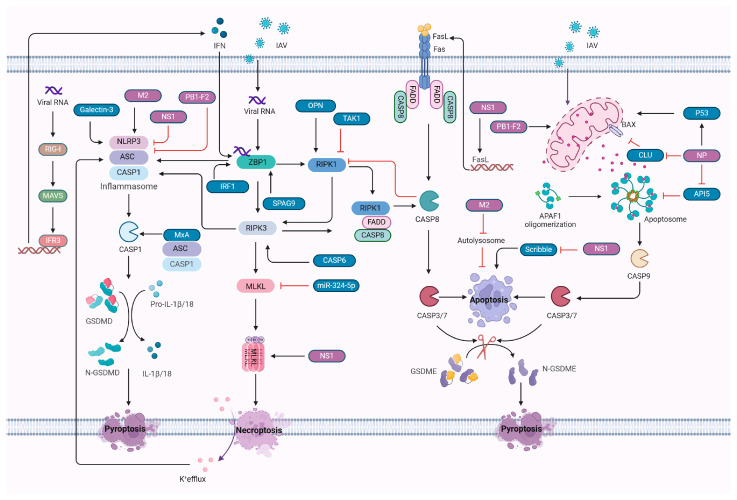
Regulation of cell death pathways by host factors and IAV proteins. During IAV infection, ZBP1 detects viral RNA, mediating apoptosis, necroptosis, and pyroptosis, with contributions from various host factors and viral proteins. PB1-F2, NP, and M2 promotes caspase-3/7-mediated apoptosis by modulating host proteins, while NS1 displays dual roles through distinct pathways. TAK1 downregulates extrinsic apoptosis by inhibiting RIPK1 activity. Activated caspase-3 promotes GSDME-mediated pyroptosis. Activated caspase-8 suppresses necroptosis by cleaving RIPK1. Host proteins caspase-6 and OPN, along with NS1, facilitate necroptosis, whereas TAK1 and the non-coding RNA miR-324-5p inhibit this process. Potassium efflux resulting from necroptosis activates the NLRP3 inflammasome, which promotes pyroptosis. M2, MxA and Galectin-3 enhance inflammasome activity, facilitating GSDMD-mediated pyroptosis, while PB1-F2 and NS1 inhibit pyroptosis through suppression of inflammasome activation. Additionally, viral RNA activates the RIG-I-MAVS pathway, leading to interferon production that upregulates ZBP1. ZBP1 orchestrates these three cell death modalities during influenza A virus infection, positively regulated by IRF1 and SPAG9. Overall, these pathways underscore the complex interplay between viral and host factors in regulating cell death during infection.

The virus also inhibits apoptosis to extend the survival duration of infected cells, allowing adequate time for viral replication (Figure 2). The non-structural protein NS1 plays a key role in this process by suppressing the host’s type I interferon (IFN) response, enabling the virus to evade immune detection [26]. Additionally, NS1 binds to the pro-apoptotic protein Scribble, effectively blocking its apoptotic function, thereby extending viral persistence in host cells [27]. Mutations in NS1′s PDZ-binding motif disrupt this anti-apoptotic function, which significantly reduces viral replication [27]. Notably, the NS1 protein from the H5N1 strain has been demonstrated to upregulate FasL mRNA expression, further promoting apoptosis [28]

IAV not only manipulates intrinsic pathways to regulate apoptosis but also utilizes host proteins to activate extrinsic apoptotic pathways. Recent studies have identified ZBP1 (Z-DNA binding protein 1), also known as DAI (DNA-dependent activator of interferon-regulatory factors), as a key nucleic acid sensor crucial for regulating cell death and innate immunity [29]. ZBP1 contains Zα domains that specifically bind to left-handed double-helical RNA structures (Z-RNAs) and a receptor-interacting homotypic interaction motif (RHIM), which is essential for interacting with other RHIM-containing mediators. ZBP1 initiates cell death signaling pathways during influenza infection by recognizing viral Z-RNAs [29,30]. In mouse embryonic fibroblasts (MEFs), ZBP1 interacts with receptor-interacting protein kinase 3 (RIPK3) through its RHIM domain, which subsequently recruits receptor-interacting protein kinase 1 (RIPK1). This interaction activates caspase-8 via the ZBP1-RIPK3-RIPK1-FADD-caspase-8 axis, mediating RIPK3-dependent, RIPK1 kinase-independent apoptosis [31,32]. Conversely, in alveolar epithelial cells type I (LET1) infected with IAV, ZBP1 activation can recruit RIPK1, FADD, and caspase-8, thereby facilitating RIPK3-independent, RIPK1 kinase-dependent apoptosis through the ZBP1-RIPK1-FADD-caspase-8 pathway [33]. ZBP1 knockout completely abrogates IAV-mediated caspase-3 cleavage and subsequent apoptosis, highlighting the critical role of ZBP1 in IAV-induced apoptosis.

A deeper understanding of how IAV manipulates apoptotic pathways could facilitate the development of targeted therapeutic strategies aimed at mitigating influenza-induced cell death and enhancing antiviral responses.

## 3. Necroptosis Mechanisms and Regulation

**Figure 3:**
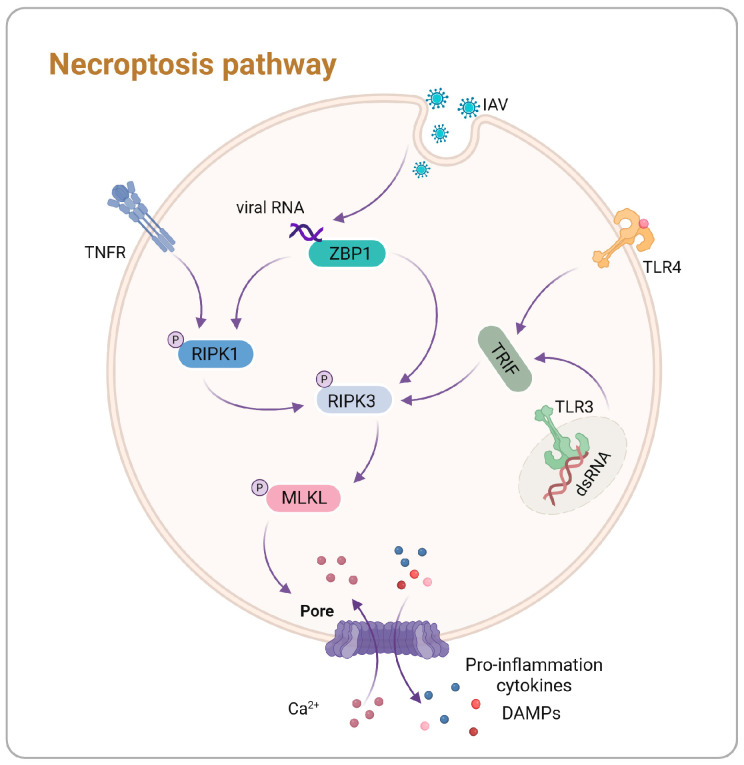
Mechanisms of necroptosis pathways. TNF-α signals through the TNFR receptor to activate RIPK1, which subsequently recruits and activates RIPK3. Viral RNA is recognized by ZBP1, leading to the activation of RIPK1 and the subsequent recruitment and activation of RIPK3, or directly activating RIPK3 through ZBP1. TLR3 and TLR4 activate RIPK3 via the adaptor protein TRIF. Activated RIPK3 phosphorylates MLKL, resulting in MLKL oligomerization and translocation to the plasma membrane, where it forms pores that mediate calcium influx and release pro-inflammatory cytokines and DAMPs.

Necroptosis is a pro-inflammatory form of programmed cell death critical in various physiological and pathological contexts, including immune responses, tumor progression, pathogen infections, and neurodegenerative diseases [34,35,36,37,38]. Necroptosis can be triggered by various factors, including toll-like receptor (TLR)-3 and TLR-4 agonists, TNFα, and viruses like IAV, through distinct mechanisms (Figure 3). Necroptosis is mediated by RIPK1, RIPK3, and mixed lineage kinase domain-like protein (MLKL). This pathway results in organelle swelling, early membrane rupture, subsequent cell lysis, ultimately leading to the release of pro-inflammatory cytokines (Table 1) [39]. Understanding these components and their interactions provides insight into necroptosis and its modulation by pathogens like the IAV.

### 3.1. Mechanistic Insights into Necroptosis

Necroptosis features a well-defined signaling cascade, with RIPK1 and RIPK3 playing central roles. RIPK1 comprises an N-terminal kinase domain, a C-terminal death domain (DD), and an intermediate RHIM domain. The activation of RIPK1 is intricately regulated through phosphorylation and ubiquitination [40]. Similarly, RIPK3 contains a RHIM domain, and upon activation, it phosphorylates MLKL. Phosphorylated MLKL oligomerizes and translocates to the plasma membrane, forming pores that disrupt membrane integrity and osmotic balance, ultimately leading to cell swelling and cell death [41,42].

Research on necroptosis has predominantly focused on the tumor necrosis factor (TNF)-mediated pathway. Upon binding of TNF to its receptor, TNFR1, a signaling complex, known as complex I, is formed on the cytoplasmic side. This complex consists of TRADD, TRAF2, RIPK1, the linear ubiquitin chain assembly complex (LUBAC), and E3 ubiquitin ligases cIAP1 and cIAP2. Under normal conditions, complex I activates the nuclear factor-kappa B (NF-κB) and mitogen-activated protein kinase (MAPK) signaling pathways, promoting survival signals and pro-inflammatory responses [43]. When cIAP1 and cIAP2 are inhibited or dysfunctional, RIPK1 undergoes deubiquitination, resulting in the dissociation of complex I from the plasma membrane. This leads to the assembly of complex II, consisting of RIPK1, FADD, and caspase-8, which activates caspase-8 and initiates apoptosis [44]. Conversely, when caspase-8 activity is inhibited, RIPK1 interacts with RIPK3 through its RHIM domain, forming the necrosome and triggering mutual phosphorylation [42,45]. Subsequently, RIPK3 phosphorylates MLKL, promoting its oligomerization and forming pores in the plasma membrane, ultimately executing necroptosis.

ZBP1 serves as a crucial nucleic acid sensor that activates the necroptosis by recognizing viral RNA through its Zα domain. It then recruits downstream signaling molecules, such as RIPK3, via its RHIM domain, facilitating the mediation of necroptosis [46]. Specific DNA viruses, including herpesviruses, activate ZBP1 through the double-stranded DNA, while certain RNA viruses, such as the IAV, engage ZBP1 through RNA recognition, ultimately leading to necroptosis [47,48,49].

Moreover, stimulation of TLR4 or TLR3 facilitates the formation of necrosomes through the adaptor protein TRIF, which contains RHIM domains, leading to RIPK3-dependent necroptosis [50,51]. The role of RIPK1 is context-dependent and influenced by the cellular environment [52,53]. These mechanisms underscore the various strategies employed by both viruses and host cells to modulate necroptosis in response to viral infections.

### 3.2. Influenza A Virus Regulation of Necroptosis

IAV replication primarily occurs in respiratory epithelial cells, alveolar macrophages, and monocyte cells, influenced by host factors [54]. Notably, the key proteins involved in mediating necroptosis, ZBP1 and RIPK3, have not been identified in avian species, and commonly used cell lines in biomedical research, such as HeLa and A549, lack RIPK3 expression [48,55]. In contrast, mouse embryonic fibroblasts (MEFs) have been shown to express both ZBP1 and RIPK3. ZBP1 recognizes viral Z-RNA and subsequently interacts with and activates RIPK3, initiating a necroptotic response during IAV infection [33]. This process can occur independently of RIPK1, as ZBP1 directly triggers RIPK3, which subsequently phosphorylates MLKL and induces necroptosis [31]. In influenza-infected alveolar epithelial type I (LET1) cells, ZBP1 is capable of activating RIPK1 [33]. This interaction facilitates the engagement of RIPK3, leading to a RIPK1-dependent necroptosis [33]. The dual capacity of ZBP1 to activate necroptosis through both RIPK1-dependent and RIPK1-independent pathways underscores the virus’s ability to manipulate host cell death mechanisms, contributing to tissue damage and enhancing viral pathogenesis.

Additionally, caspase-6 has been shown to enhance necroptosis during influenza infection by promoting the RHIM-dependent interaction between ZBP1 and RIPK3, thereby facilitating both necroptosis and apoptosis [56]. The viral protein NS1 of IAV further contributes to promoting necroptosis by enhancing MLKL oligomerization and its translocation to the membrane [57]. Osteopontin (OPN), induced by the virus, enhances the mRNA levels of RIPK1, RIPK3, and MLKL, thereby promoting necroptosis in macrophages [58]. Furthermore, recent research has identified that microRNAs (miRNAs) also play a role in regulating IAV-induced necroptosis. Dou et al. reported that miR-324-5p specifically targets the 3′ UTR of human MLKL, and the interferon-mediated reduction of miR-324-5p alleviates its inhibition of human MLKL mRNA, thus promoting necroptosis in IAV-infected cells and limiting viral replication [59] (Figure 2).

## 4. Pyroptosis Mechanism and Regulation

**Figure 4:**
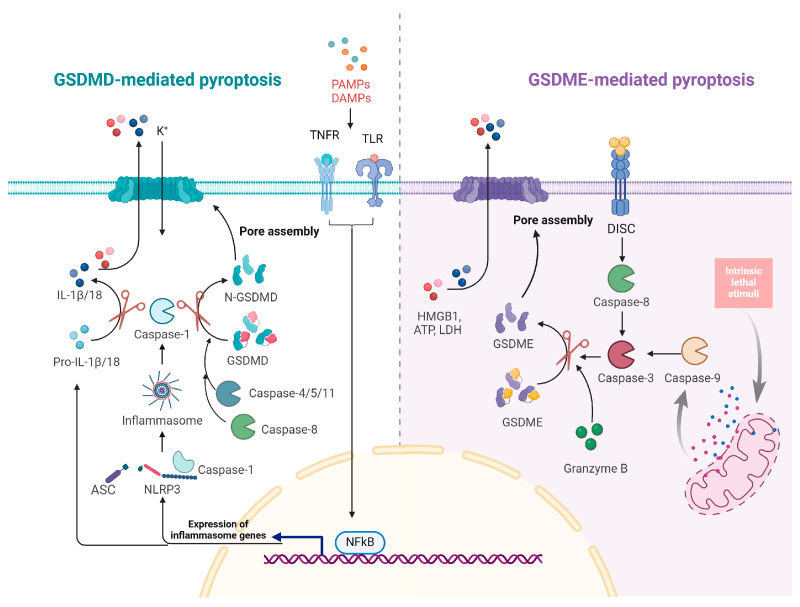
Mechanisms of pyroptosis activation mediated by GSDMD and GSDME. External stimuli activate pattern recognition receptors, leading to NF-κB activation and promoting the transcription of pro-inflammatory cytokines (pro-IL-1β, pro-IL-18). This process facilitates the assembly of the NLRP3 inflammasome and activates caspase-1. Activated caspase-1 cleaves GSDMD, resulting in pore formation in the plasma membrane that disrupts osmotic balance and induces cell swelling. Additionally, caspases-8, -4, -5, and -11 can directly cleave GSDMD in response to various stimuli. Various stimuli activate caspase-9 and caspase-8, which subsequently activate caspase-3, leading to the cleavage of GSDME. Furthermore, granzyme B can also cleave GSDME directly, resulting in pore formation and the execution of pyroptosis.

Pyroptosis is a highly inflammatory form of programmed cell death primarily mediated by gasdermin proteins [60]. Gasdermin D (GSDMD) and Gasdermin E (GSDME) are the most extensively studied, with well-defined mechanisms driving pyroptosis (Figure 4). Other gasdermin family members, such as Gasdermin A, Gasdermin B, and Gasdermin C, also have the potential to induce cell death. Gasdermins typically possess two conserved domains: an N-terminal domain responsible for pore formation in the plasma membrane and a C-terminal domain that regulates this activity [61,62]. Pyroptosis is characterized by organelle swelling and DNA fragmentation in affected cells. The formation of pores by the N-terminal domain leads to cell lysis and the subsequent release of intracellular contents, which triggers a robust inflammatory response (Table 1) [63]. Pyroptosis is associated with various pathological conditions, including infectious diseases, autoimmune disorders, cancer, and neurodegenerative diseases [64].

### 4.1. Mechanistic Insights into Pyroptosis

Pyroptosis was initially characterized as a form of cell death dependent on caspase-1, which is triggered through the activation of inflammasomes [65,66]. Inflammasomes are multiprotein complexes that consist of a receptor protein from the NLR (nucleotide-binding oligomerization domain-like receptors) or ALR (AIM-like receptors) families, an adaptor protein known as ASC (apoptosis-associated speck-like protein containing a CARD), and caspase-1 [65,66].

Currently, there are several known pattern recognition receptor (PRR) members involved in inflammasome formation, including NLRP3, NLRP1, NLRC4/NAIP, AIM2, Pyrin, and NLRP6 [67]. Activation of inflammasomes, such as NLRP3 or AIM2, occurs in response to pathogen-associated molecular patterns (PAMPs) or damage-associated molecular patterns (DAMPs) [67]. This process engages NF-κB, which subsequently promotes the transcription of pro-inflammatory cytokines, including pro-interleukin-1β (pro-IL-1β) and pro-interleukin-18 (pro-IL-18).

The classical pyroptotic pathway begins with inflammasome assembly and subsequent activation of caspase-1. Activated caspase-1 cleaves GSDMD at the Asp275, generating the active N-terminal fragment [60]. In humans, caspase-4 and caspase-5, as well as caspase-11 in mice, can directly bind intracellular LPS; activation of these caspases also leads to the cleavage of GSDMD [68,69]. In the presence of TAK1 inhibitors, Yersinia or LPS can induce RIPK1- and caspase-8-dependent cleavage of GSDMD [17,70]. The N-terminal fragment of GSDMD oligomerizes and integrates into the plasma membrane, forming non-selective pores that disrupt cellular homeostasis. These pores disrupt osmotic balance, causing cell swelling and pyroptotic cell death [62,71]. Additionally, caspase-1 processes pro-IL-1β and pro-IL-18, facilitating their release through the pores formed by GSDMD [62,71].

Chemotherapy induces a distinct form of non-canonical pyroptosis mediated by caspase-3, which relies on the expression of GSDME. Initially, chemotherapeutic agents activate and oligomerize BAK and BAX proteins on the mitochondrial outer membrane, resulting in mitochondrial outer membrane permeabilization (MOMP) and the release of cytochrome c [72]. This process activates caspase-9, which subsequently activates effector caspase-3. Additionally, caspase-3 can be further activated by caspase-8 via death receptor signaling pathways. Once activated, caspase-3 cleaves GSDME at Asp270, generating C-terminal and N-terminal fragments. The N-terminal fragment translocates to the plasma membrane, forming pores that trigger pyroptosis [73]. Recent findings also indicate that granzyme B can cleave GSDME at the same site as caspase-3, enhancing the pyroptotic response [74].

### 4.2. Influenza A Virus Regulation of Pyroptosis

IAV manipulates host pyroptotic pathways to enhance its pathogenicity. The regulation of IAV-induced pyroptosis is mediated by several host factors that control both immune responses and cell death pathways. During IAV infection, ZBP1 is critical for activating the NLRP3 inflammasome and facilitating IL-1β and IL-18 secretion in bone marrow-derived macrophages (BMDMs) [75]. Notably, ZBP1-deficient BMDMs exhibit significantly reduced pyroptosis and cytokine production [75]. Furthermore, in BMDMs lacking type I interferon receptor 1 (IFNAR1), ZBP1 expression and the induction of pyroptosis are completely inhibited, highlighting the necessity of type I interferon signaling for ZBP1-dependent pyroptosis activation during IAV infection [76]. Additionally, ZBP1-induced pyroptosis in BMDMs during IAV infection is modulated by the RIG-I-MAVS and TLR signaling pathways [77]. The DNA sensor IFI16 also contributes to the immune response by recognizing viral RNA and triggering pro-inflammatory cytokines like IFN-β [78]. Potassium efflux is a recognized trigger for NLRP3 inflammasome activation. During IAV infection, MLKL-mediated necroptosis causes plasma membrane rupture, resulting in potassium efflux that activates the NLRP3 inflammasome in BMDMs [79]. This amplifies the antiviral response, suppressing viral replication while promoting pyroptosis in infected cells. In summary, IAV infection enhances interferon production via the RIG-I-MAVS pathway, with IFI16 boosting this signaling, resulting in the activation of ZBP1. ZBP1-mediated necroptosis induces potassium efflux, indirectly activating the NLRP3 inflammasome and promoting pyroptosis. This underscores the complexity of host responses in regulating pyroptosis and balancing immune defense with tissue integrity during influenza infections.

Additionally, NLRP3 is primarily expressed in macrophages rather than in normal airway epithelial cells [80,81], suggesting that alternative inflammasome sensors may mediate pyroptosis during IAV infection. For instance, myxoma resistance protein 1 (MxA) interacts with ASC to promote inflammasome formation in human respiratory epithelial cells [82]. Galectin-3 enhances H5N1-induced inflammation by facilitating NLRP3 inflammasome assembly and promoting IL-1β secretion [83], while the IAV matrix protein M2 further stimulates inflammasome activation and caspase-1-mediated cytokine release [84].

However, IAV has developed countermeasures to inhibit pyroptosis (Figure 2). For example, Cheung et al. reported that the PB1-F2 protein of H7N9 selectively suppresses NLRP3 inflammasome activation and IL-1β production by disrupting the interaction between NLRP3 and MAVS [85]. NEK7, a member of the mammalian NIMA-related kinases (Neks), is a crucial NLRP3-binding protein that mediates NLRP3 inflammasome assembly and activation [86]. Boal-Carvalho et al. found that PB1-F2 binds directly to NLRP3, inhibiting its interaction with NEK7 and thereby suppressing pyroptosis and IL-1β release [87]. Similarly, NS1 protein inhibits NLRP3/ASC speck formation, attenuating inflammasome activation [88].

Beyond GSDMD, other gasdermins, such as GSDME, are activated through distinct pathways [73,89]. Influenza infection activates caspase-3, which cleaves GSDME, leading to pyroptosis and cytokine release. This pathway is particularly pronounced during H7N9 infection, exacerbating lung tissue damage and contributing to cytokine storms that worsen the inflammatory response [90]. Guy et al. reported that IAV induces caspase-3 activation via a PKR-dependent pathway, leading to GSDME cleavage and pyroptosis in human epithelial cells [91]. The influenza-induced activation of GSDME further exacerbates cell death and inflammation, highlighting the virus’s ability to exploit different forms of pyroptosis to its advantage.

Interestingly, several studies have demonstrated the involvement of Ninjurin 1 (NINJ1) in plasma membrane rupture during pyroptosis [92]. Each NINJ1 subunit consists of two amphipathic helices (α1 and α2) and two transmembrane helices (α3 and α4), forming a chain of subunits mainly through interactions among the transmembrane helices and α1. Notably, the kinked structures of α3 and α4, along with the glycine residues, are crucial for its function [93].

## 5. Activation and Interplay of Cell Death Pathways in Influenza A Virus Infection

IAV infection triggers a complex network of cell death pathways in host cells, including apoptosis, necroptosis, and pyroptosis. These pathways do not function independently; rather, they are interconnected through intricate cross-talk involving sequential activations and mutual inhibition, regulated by both viral proteins and host factors (Figure 2).

Infection initiates as hemagglutinin (HA) binds to sialic acid receptors on the host cell surface, facilitating viral entry and setting the stage for cell death pathway activation. Once inside the cell, viral components are detected by PRRs, including RIG-I and MDA5, which trigger antiviral responses and cellular stress [94]. This process activates pathways such as NF-κB and IRF3, promoting interferon production and pro-inflammatory cytokines to amplify immune responses, specifically upregulating ZBP1 expression through RIG-I-MAVS signaling [77].

As the virus replicates and accumulates within the cell, ZBP1 recognizes viral Z-RNA, leading to the initiation of multiple cell death pathways [29,30]. ZBP1-mediated apoptosis, necroptosis, and pyroptosis are further enhanced by IRF1 and SPAG9 [95,96]. The virus activates caspase-8 through ZBP1, triggering extrinsic apoptosis and cleaving RIPK1 to inhibit necroptosis [31,32,33,97]. Concurrently, IAV activates intrinsic apoptosis through mitochondrial disruption, with viral proteins such as NP and PB1-F2 modulating apoptosis through diverse mechanisms (Table 2). This regulatory network is further refined as activated caspase-3 cleaves GSDME to trigger pyroptosis, illustrating a pathway hierarchy where apoptosis can suppress necroptosis while facilitating pyroptosis [90].

Host factors TAK1 and miR-324-5p inhibit necroptosis by downregulating RIPK1 and MLKL [33,59]. In contrast, caspase-6 and viral NS1 promote necroptosis by enhancing interactions between RIPK3 and ZBP1, as well as facilitating MLKL oligomerization [56,57]. Potassium ion efflux resulting from necroptotic cell rupture activates the NLRP3 inflammasome, which subsequently activates caspase-1, releasing IL-1β to enhance immune responses [79]. The ion channel protein M2 also promotes inflammasome activation and pyroptosis, while PB1-F2 and NS1 act as inhibitors [84,85,87,88]. Pyroptosis releases cellular contents, creating an inflammatory feedback loop that influences apoptosis and necroptosis in surrounding cells, further promoting cell death.

This dynamic network highlights the role of viral and host factors in coordinating cross-talk between cell death pathways during influenza virus infection, shaping the host’s response and impacting disease progression.

## 6. PANoptosis Mechanisms and Regulation with Influenza A Virus

**Figure 5:**
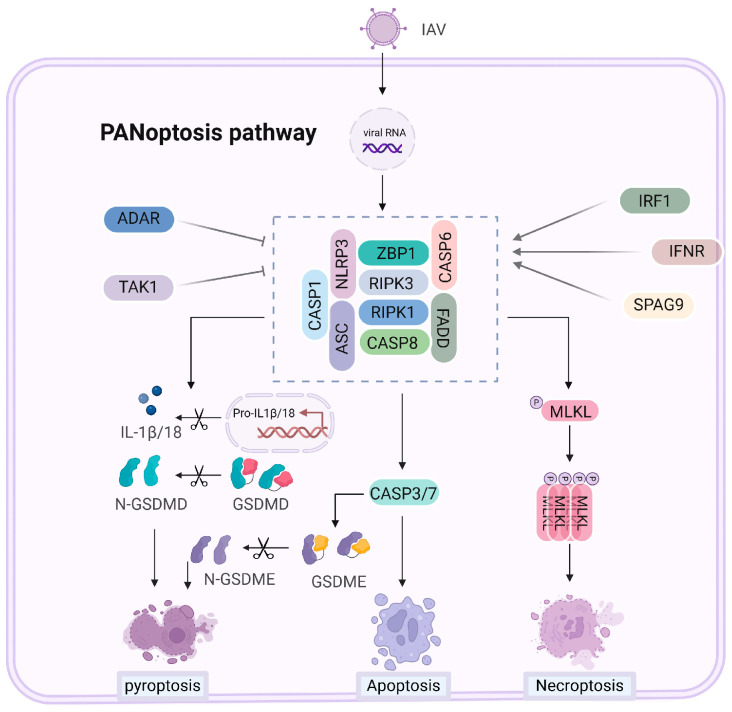
Mechanisms of PANoptosis activation during IAV infection. This diagram illustrates the PANoptosome complex during IAV infection, highlighting the role of ZBP1 in detecting viral RNA to activate pyroptosis, apoptosis, and necroptosis, thereby orchestrating a multifaceted immune response. ZBP1 detects viral RNA and initiates the PANoptotic response, leading to the activation of pyroptosis (via GSDMD and GSDME), apoptosis (via caspases-3 and -7), and necroptosis (via MLKL). The interaction between ZBP1 and SPAG9 enhances PANoptosome assembly, while ZBP1 expression is regulated by IRF1 and IFNAR, both of which modulate immune responses. Inhibition of TAK1 promotes RIPK1-dependent cell death. Additionally, ADAR1 prevents spontaneous activation of ZBP1, maintaining cellular homeostasis and mitigating the risk of excessive PANoptosis.

PANoptosis is an inflammatory cell death mechanism that integrates pyroptosis, apoptosis, and necroptosis [2,98]. Unlike conventional cell death pathways that operate independently, PANoptosis is characterized by the simultaneous activation of these mechanisms, providing a comprehensive immune response during pathogenic infections, such as IAV infection. Figure 5 illustrates the components of the PANoptosome complex and its regulation during IAV infection.

### 6.1. Mechanisms of PANoptosis Activation

During IAV infection, the PANoptosome complex forms by recruiting essential signaling molecules such as ZBP1, RIPK1, RIPK3, NLRP3, ASC, FADD, and caspases, including caspase-1, caspase-8, and sometimes caspase-6 [2,56,98]. ZBP1 plays a pivotal role in detecting viral RNA and viral proteins like NP and PB1-F2, which initiate the PANoptosis [75]. Its expression is modulated by factors such as IRF1 and IFNAR, enhancing its role during IAV infection. This leads to the activation of GSDMD and GSDME-mediated pyroptosis, caspase-3 and caspase-7-mediated apoptosis, and MLKL-mediated necroptosis simultaneously. By coordinating multiple cell death pathways, PANoptosis ensures the efficient elimination of infected cells while triggering a robust inflammatory response.

### 6.2. Influenza A Virus Regulation of PANoptosis

The key to PANoptosis activation during IAV infection is the ZBP1 protein, specifically its Zα2 domain, which is critical for detecting IAV and assembling the PANoptosome. In addition to initiating PANoptosis, the Zα2 domain activates the NLRP3 inflammasome during IAV infection, further enhancing inflammation [99]. Another regulatory component is sperm-associated antigen 9 (SPAG9), which interacts with ZBP1 to facilitate the DAI/SPAG9/JNK signaling pathway [96]. This interaction strengthens the connection between RIPK1, RIPK3, and ZBP1, promoting PANoptosome formation and driving IAV-induced cell death.

Interestingly, Kuriakose et al. demonstrated the role of IRF1 in regulating ZBP1 expression, independently of type I interferon signaling. In response to IAV infection, IRF1-konckout macrophages exhibit lower induction of ZBP1 protein compared to wild-type cell, which correlates with reduced activation of PANoptosis [95]. Additionally, in the presence of a TAK1 inhibitor combined with LPS stimulation, IRF1-deficient cells show a decrease in PANoptosis compared to wild-type cells [100]. IFNAR serves as the receptor for type I interferons (IFN-α/β). Its signaling pathway activates interferon regulatory factor 1 (IRF1) and other interferon-stimulated genes, including ZBP1, thereby promoting PANoptosis, especially in the context of viral infections [75]. Furthermore, adenosine deaminase acting on RNA (ADAR1) inhibits the spontaneous activation of the left-handed Z-DNA sensor ZBP1. ZBP1 is implicated in the embryonic lethality observed in ADAR knockout mice and is associated with increased early mortality and intestinal cell death in mice with defects in ADAR and MAVS expression [101]. TGF-β-activated kinase 1 (TAK1) is a crucial kinase that regulates both pro-survival and pro-inflammatory signaling pathways, functioning upstream of NF-κB and MAPK signaling. Inhibition or loss of TAK1 enhances the activation of RIPK1, resulting in increased RIPK1-dependent apoptosis and necroptosis during IAV infection in LET1 cells [33]. Additionally, TAK1 inhibition—by either the YopJ protein from Yersinia or TAK1 inhibitors in LPS-treated BMDMs, promotes RIPK1-mediated GSDMD activation and pyroptosis [70,102,103]. These findings underscore the complex interplay between these proteins in regulating immune responses and cell death pathways during viral infections.

## 7. Cell Death and Inflammatory Response in IAV Infection

During IAV infection, programmed cell death pathways have both protective and harmful effects on the immune response. While these pathways are essential for clearing the virus, excessive activation can lead to a cytokine storm, a hyperinflammatory condition characterized by elevated circulating cytokines and subsequent widespread tissue damage. Infected respiratory epithelial cells, key targets of IAV, drive pro-inflammatory responses necessary for antiviral defense, yet this can also lead to significant collateral damage, exacerbating the severity of the infection [104]. This balance is critical, as an exaggerated immune response can result in severe pathology, such as diffuse alveolar damage and fibrotic repair [105].

In respiratory virus infections, a heightened immune response, particularly with excessive release of IL-1, IL-6, and TNF-α, can escalate systemic inflammation, potentially causing multi-organ failure. The early surge of IL-1, followed by IL-6, plays a pivotal role in driving hyperinflammation. Therapeutic interventions using IL-1β receptor antagonists or neutralizing antibodies have demonstrated effectiveness in mitigating inflammation during severe H1N1 infections [106]. This highlights the therapeutic potential of modulating inflammation to prevent tissue damage while still allowing effective antiviral responses.

PANoptosis plays a pivotal role in the pathology associated with the cytokine storm observed in IAV and SARS-CoV-2 infections. ZBP1 knockout mice infected with IAV demonstrate significant reductions in key inflammatory cytokines, emphasizing ZBP1’s critical role in mediating immune responses [107]. However, ZBP1 deficiency also increases susceptibility to IAV, impairing viral clearance and survival [32]. This dual role exemplifies how programmed cell death is vital for viral elimination but can lead to severe outcomes if uncontrolled. Notably, TNF-α and IFN-γ cotreatment mimics the severe cases of SARS-CoV-2, and neutralizing antibodies against these cytokines effectively protect mice from SARS-CoV-2 infection [108]. In IAV infections, K63-linked polyubiquitination of ZBP1 by TRIM34 enhances RIPK3 recruitment, facilitating necroptotic signaling [109]. Mice lacking ZBP1 or TRIM34 exhibit higher viral loads and lower cytokine levels, resulting in prolonged survival [109]. Elevated levels of ZBP1 correlate with poor outcomes in COVID-19 patients, where ZBP1-induced PANoptosis and the resultant cytokine storm hinder effective treatment [110].

Necroptosis plays a complex role in IAV infections. Nogusa et al. reported that RIPK3 restricts viral titers and lung damage, thereby providing a protective effect [31]. Conversely, uncontrolled RIPK3 activation in cIAP2-deficient mice leads to severe tissue injury and elevated mortality; notably, co-deletion of RIPK3 can rescue these mice from fatal outcomes [111]. MLKL deficiency offers protection against lethal doses of both PR8 [112] and A/California/7/2009 [113] virus strains. In MLKL-deficient mice, lung epithelial disruption is reduced, leading to diminished neutrophil infiltration and improved survival [30]. These findings indicate that while necroptosis serves as a protective mechanism, its dysregulation can cause significant tissue damage. Moreover, reduced cIAP2 expression is associated with severe acute respiratory distress syndrome (ARDS) in H7N9 infections [114].

The NLRP3 inflammasome and pyroptosis are critical in host defense, promoting the release of pro-inflammatory cytokines [115]. During IAV infection, loss of DDX3X impairs NLRP3 activation and type I IFN production, resulting in severe lung damage and reduced survival [116]. Consistently, NLRP3-deficient mice exhibit increased susceptibility to PR8 infection [117]. However, excessive NLRP3 activation can exacerbate inflammation in severe cases. Mice lacking caspase-1 or NLRP3 produce fewer pro-inflammatory cytokines and exhibit reduced morbidity and mortality during H7N9 infections [118]. In H5N1-infected macaques, IL-1β and GSDMD activation mediates pyroptosis and intense inflammatory responses [119]. In GSDMD-deficient mice infected with H3N2, inhibition of pyroptosis results in prolonged survival [120]. Similarly, GSDME knockout prevents excessive lung inflammation in H7N9-infected mice [90]. These findings indicate that while pyroptosis is essential for immune defense, its regulation is crucial to prevent tissue damage caused by unchecked inflammation.

Recent studies highlight the therapeutic potential of targeting apoptosis to mitigate viral pathogenesis across various infections, including MERS-CoV, SARS-CoV, and coxsackievirus infections [121,122]. Inhibiting MERS-CoV-induced apoptosis with the PERK inhibitor reduces lung damage, while the intrinsic apoptosis inhibitor z-LEHD-fmk significantly improves survival rates in infected mice [121]. Similarly, Wang et al. found that targeting PERK-induced apoptosis via TRIM29 deficiency or the PERK inhibitor GSK2656157 alleviates myocarditis and prolongs survival in mice infected with coxsackievirus B3 (CVB3) [122]. Moreover, FADD and RIPK3 double-knockout mice exhibited increased susceptibility to IAV infection compared to wild-type or RIPK3-knockout counterparts, emphasizing the critical role of FADD-mediated apoptosis in controlling IAV-induced mortality [123]. These findings underscore that apoptosis can modulate immune responses effectively.

The influence of virus-induced cell death on disease progression is significantly affected by various factors, including viral inoculation dose, individual variability among animals, and strain differences [124]. For instance, H7N9 is linked to severe hemorrhagic manifestations, contrasting with the relatively milder effects of PR8 (H1N1). At elevated inoculation doses, key proteins such as ZBP1 and NLRP3 may exacerbate immunopathology, leading to increased mortality rates. This highlights the dual role of these proteins in viral infections and emphasizes the need for targeted therapeutic strategies to mitigate the adverse effects of excessive cell death while preserving effective antiviral responses.

## 8. Therapeutic Strategies Targeting Cell Death in Influenza A Virus Infection

IAV-induced cell death significantly impacts both viral pathogenesis and immune-mediated tissue damage, making it a promising target for therapeutic intervention. Multiple cell death pathways—including pyroptosis, necroptosis, and apoptosis—are activated during IAV infection. Targeting these pathways through inhibitors and small molecule interventions holds potential for mitigating the adverse effects of influenza, particularly in severe cases (Table 3).

### 8.1. Small Molecule Inhibitors of Cell Death Pathways

#### 8.1.1. Inhibition of NLRP3-Mediated Pyroptosis

Targeting pyroptosis is a promising therapeutic strategy for mitigating acute respiratory distress syndrome (ARDS) in severe influenza cases. Inhibiting NLRP3 effectively reduces inflammation and issue damage across various viral infections. For example, MCC950, a selective NLRP3 inhibitor, directly interacts with the Walker B motif within the NACHT domain, thereby blocking ATP hydrolysis and inhibiting NLRP3 activation and inflammasome formation [125]. MCC950 has been reported to inhibit airway neutrophil infiltration, IL-1β secretion and lung inflammation induced by PB1-F2 peptides derived from both PR8 and H7N9 strains in mouse models [126]. In addition, MCC950 effectively inhibits IAV-induced NLRP3 inflammasome activation in differentiated human bronchial epithelial cells, reducing airway inflammation and improving survival in COPD rat models [127]. This effect extends to SARS-CoV-2, where both MCC950 and Ac-YVAD-cmk, a caspase-1 inhibitor, reduce lung injury by blocking cytokine production [128].

Cytidine monophosphate kinase 2 (CMPK2) is a crucial enzyme that provides deoxyribonucleotides for mitochondrial DNA synthesis and facilitates the activation of the NLRP3 inflammasome through the generation of cytosolic oxidized mtDNA [145]. CMPK2 inhibitors, such as Z25 and Z08, show potential in reducing RSV-induced pulmonary inflammation, indicating broader applicability in IAV infection [76]. VX-765, a caspase-1 inhibitor, is also promising for inhibiting pyroptosis and mitigating inflammation during virus infection [129,130]. Disulfiram, a GSDMD pore formation inhibitor, has demonstrated efficacy in reducing acute lung injury and improving survival in animal models of TRALI and SARS-CoV-2 infection [131,132]. While promising for mitigating influenza-induced lung damage, further studies are essential to confirm its therapeutic potential and examine any off-target effects in viral infections.

Respiratory viral infections, such as those caused by IAV and SARS-CoV-2, can trigger the excessive release of pro-inflammatory cytokines, notably IL-1β, IL-6, and TNF-α. This cytokine surge significantly contributes to the cytokine storms associated with severe tissue damage in these infections. Anti-inflammatory therapies targeting IL-1 and IL-6 have shown promise in mitigating these cytokine storms in severe influenza infections, as demonstrated in clinical trials for related respiratory conditions, such as COVID-19. Agents like canakinumab, an IL-1β monoclonal antibody, and anakinra, a recombinant IL-1 receptor antagonist, have exhibited clinical efficacy in controlling severe inflammation in SARS-CoV-2-associated pneumonia [133,134,135]. Notably, during influenza-induced exacerbation, treatment with anakinra significantly reduced neutrophil recruitment to the airways at the peak of virus-induced inflammation [136]. In acute necrotizing encephalopathy caused by H1N1 pdm09 and H1N1 2019 infections, early intervention with tocilizumab, an IL-6 receptor antagonist, has demonstrated improved clinical outcomes [137,138]. These findings underscore the potential of targeting hyperinflammatory responses in severe influenza cases; however, further studies are essential to validate the safety and efficacy of these therapies in this context.

#### 8.1.2. Inhibition of RIPK1 and RIPK3-Mediated Necroptosis

Necroptosis, mediated by RIPK1 and RIPK3, is a significant contributor to tissue damage during influenza infections. RIPK1 inhibitors, such as Necrostatin-1 (Nec-1) and its derivative Nec-1s, have demonstrated protective effects in lung injury models by inhibiting RIPK1-RIPK3 signaling and reducing inflammatory cytokines [139,140,141]. However, their clinical application is limited due to suboptimal pharmacokinetics and potential off-target effects, raising concerns about safety and efficacy in the context of viral infections.

Inhibitors targeting RIPK3, such as GSK’872, GSK’843, and GSK’840, have shown efficacy in mitigating necroptosis in preclinical models [142]. Nonetheless, the potential for these compounds to induce apoptosis in a concentration-dependent manner complicates their clinical application [146]. Notably, the RIPK3 inhibitor UH15-38 has been demonstrated to prevent IAV-induced necroptosis in alveolar epithelial cells, significantly reducing lung inflammation and mortality in H1N1 PR8 infected murine models while preserving antiviral immune response [112]. Additionally, Necrosulfonamide (NSA), an MLKL inhibitor, has exhibited protective effects in various models, indicating its potential application in necroptosis-related pathologies induced by IAV [147,148].

Future studies should focus on optimizing these inhibitors for clinical use, thereby contributing to a more integrated approach in the management of viral pathogenesis and its complications.

#### 8.1.3. Inhibition of Caspase-Mediated Apoptosis

Inhibiting caspase-mediated apoptosis represents a promising approach to reduce influenza-induced tissue damage, particularly by preventing excessive cell death in infected airway epithelial cells. For instance, the caspase inhibitor z-LEHD-fmk has demonstrated improved survival and reduced lung injury in animal models of other respiratory viruses MERS-CoV, suggesting its potential utility in influenza treatment [121].

### 8.2. Combination Therapies and Clinical Prospects

Combination therapies that simultaneously target multiple cell death pathways represent a promising strategy to enhance treatment outcomes in influenza patients. Building on the efficacy of individual inhibitors, the synergistic use of NLRP3 inhibitors with autophagy modulators may present a more comprehensive approach, effectively reducing inflammation and enhancing cellular resilience in influenza-infected tissues [149]. However, rigorous clinical validation is essential to confirm the safety and efficacy of these combinations.

Furthermore, integrating antiviral therapies with tissue-protective agents, may provide enhanced protection against influenza while reducing tissue injury. For instance, a study by Hung et al. demonstrated that a combination of clarithromycin (an antibiotic), naproxen (a COX inhibitor), and oseltamivir (a neuraminidase inhibitor) significantly reduced viral titers, shortened hospital stays, and reduced mortality rates in H3N2-infected patients compared to oseltamivir alone [143].

Additionally, delayed treatment with oseltamivir combined with sirolimus has shown effectiveness in reducing pro-inflammatory cytokines and chemokines, alleviating severe lung injury, and lowering viral titers in H1N1 pdm09 infection [144]. This combination targets the mTOR-NF-κB-NLRP3 inflammasome-IL-1β axis, representing a superior therapeutic approach compared to monotherapy with either drug. Collectively, these findings advocate for the exploration and clinical implementation of combination therapies to improve treatment efficacy in influenza and potentially other viral infections.

## 9. Conclusions and Perspectives

Host cell death pathways—including apoptosis, necroptosis, and pyroptosis—play complex roles in influenza infection by limiting viral replication and triggering immune defenses. However, excessive activation of these pathways can lead to harmful inflammation and tissue damage. Thus, future therapeutic approaches should target the precise modulation of these pathways to enhance antiviral activity while controlling inflammation.

Despite progress, there remain substantial knowledge gaps in understanding how influenza viruses manipulate host cell death mechanisms. Specifically, the interactions between viral proteins and host mediators such as ZBP1, RIPK3, and caspase-8 are not fully elucidated. Clarifying these mechanisms could reveal novel therapeutic targets, enabling the design of specific inhibitors that minimize immune interference. Additionally, identifying predictive biomarkers for cell death pathway activation would allow for personalized treatments tailored to different patient profiles.

While existing inhibitors, like Nec-1s for RIPK1, and UH15-38 for RIPK3, hold potential, they face clinical challenges, including limited efficacy, pharmacokinetic constraints, and off-target effects. Influenza virus often activates multiple cell death pathways simultaneously, creating compensatory mechanisms that undermine single-pathway inhibition. For instance, inhibiting apoptosis alone may shift cells towards alternative pathways, such as necroptosis. Addressing this complexity may require combination therapies that modulate multiple pathways concurrently.

To improve therapeutic outcomes, future drug development should prioritize combination or multi-targeted therapies. For example, co-targeting NLRP3 and caspase pathways could limit pyroptotic and apoptotic death, particularly in severe influenza cases. Additionally, selective inhibition of virus-specific interactions with host proteins, offers a promising strategy to limit pathogenic cell death without broadly suppressing immune function. Developing biomarkers to guide patient stratification and monitor responses is equally critical for enabling targeted, personalized influenza therapies.

Emerging candidates, such as MCC950, UH15-38, and Disulfiram, as well as combination regimens involving antiviral and anti-inflammatory agents, show promise in reducing both viral load and inflammation. Incorporating mTOR-NF-κB-NLRP3 pathway inhibitors may further enhance efficacy by addressing both viral replication and inflammatory damage.

Striking a balance between effective pathogen clearance and controlled inflammation will be essential for optimizing therapeutic outcomes in influenza and other viral infections. Future research should focus on refining modulators of cell death pathways, exploring combination strategies, and deepening understanding of virus-host interactions to inform precise antiviral approaches. These advancements will pave the way for safer, more effective therapies that mitigate influenza-induced pathology and improve patient prognosis.

## Figures and Tables

**Table 1:** Characteristics and Differentiation of Apoptosis, Necroptosis, and Pyroptosis.

Characteristics	Apoptosis	Necroptosis	Pyroptosis
DNA Fragmentation	Yes	No	Yes
Chromatin Condensation	Yes (pyknotic nuclei)	No	Minimal or absent
Membrane Integrity	Yes (intact until late stage)	No	No
Organelle Swelling	No	Yes	Yes
Caspase Activation	Yes (Caspase-3, -7, -9, etc.)	No (RIPK1/RIPK3/MLKL pathway)	Yes (Caspase-1, -4, -5, -11, -8, -3)
Cell Death Mechanism	Programmed	Programmed	Programmed
Inflammatory Nature	No (immunologically silent)	Yes (highly inflammatory, DAMP release)	Yes (highly inflammatory, IL-1β, IL-18 release)

**Table 2:** Mechanisms of influenza viral proteins in modulating apoptosis.

Viral Protein	Mechanism of Action	Effect on Apoptosis	References
PB1-F2	Localizes to mitochondria, induces mitochondrial membrane permeabilization	Activates intrinsic apoptotic pathway	[20,21]
M2	Interacts with ATG5/Beclin-1 complex	Inhibits autophagosome fusion, promotes apoptosis indirectly	[22]
NP	Stabilizes p53, downregulates API5	Amplifies apoptosis, reduces anti-apoptotic signals	[23,24]
NP	Reduces CLU–Bax interaction	Facilitates mitochondrial apoptosis	[25]
NS1	Inhibits type I IFN response, binds to Scribble	Suppresses apoptosis, enhances viral persistence	[26,27]
NS1 (H5N1)	Upregulates FasL mRNA expression	Promotes apoptosis	[28]

**Table 3:** Therapeutic agents targeting cell death pathways in viral infections.

Drug Name	Target Protein/ Pathway	Efficacy	References
MCC950	NLRP3	Reduces inflammation and lung injury in models of IAV and SARS-CoV-2.	[125,126,127,128]
Z25, Z08	CMPK2	Reduces pulmonary inflammation in RSV and potentially IAV.	[76]
VX-765	Caspase-1	Inhibits pyroptosis and reduces inflammation during viral infections.	[129,130]
Disulfiram	GSDMD	Mitigates acute lung injury and improves survival in TRALI and SARS-CoV-2 models.	[131,132]
Canakinumab	IL-1β	Controls severe inflammation in pneumonia associated with SARS-CoV-2.	[133]
Anakinra	IL-1 Receptor	Reduces severe inflammatory responses in SARS-CoV-2 and IAV infection.	[134,135,136]
Tocilizumab	IL-6 Receptor	Improves clinical outcomes in acute necrotizing encephalopathy from H1N1 virus.	[137,138]
Necrostatin-1, Nec-1s	RIPK1	Protects against lung injury by inhibiting RIPK1-RIPK3 signaling.	[139,140,141]
GSK’872, GSK’843, GSK’840	RIPK3	Effective in mitigating necroptosis in preclinical models.	[142]
Necrosulfonamide	MLKL	Exhibits protective effects in necroptosis-related pathologies.	[112]
z-LEHD-fmk	Caspase-9	Improves survival and reduces lung injury in respiratory virus models.	[121]
Clarithromycin + Naproxen + Oseltamivir	Antiviral + anti-inflammatory	Reduces viral titers and mortality in H3N2-infected patients.	[143]
Oseltamivir + Sirolimus	mTOR-NF-κB-NLRP3	Reduces pro-inflammatory cytokines and alleviates lung injury in H1N1 pdm09 infection.	[144]

## Data Availability

Not applicable.
